# The Evolutionary Processes of Canine Coronaviruses

**DOI:** 10.1155/2011/562831

**Published:** 2011-07-07

**Authors:** Annamaria Pratelli

**Affiliations:** Department of Public Health and Animal Sciences, University of Bari, 70010 Valenzano, Bari, Italy

## Abstract

Since the first identification of the virus in 1971, the disease caused by canine coronavirus (CCoV) has not been adequately investigated, and the role that the virus plays in canine enteric illness has not been well established. Only after the emergence in 2002 of SARS in human has new attention been focused on coronaviruses. As a consequence of the relatively high mutation frequency of RNA-positive stranded viruses, CCoV has evolved and, with the biomolecular techniques developed over the last two decades, new virus strains, serotypes, and subtypes have been identified in infected dogs. Considering the widespread nature of CCoV infections among dog populations, several studies have been carried out, focusing upon the epidemiological relevance of these viruses and underlining the need for further investigation into the biology of CCoVs and into the pathogenetic role of the infections. This paper reports the evolutionary processes of CCoVs with a note onto recent diagnostic methods.

## 1. Coronaviruses: Genome and Structure

Coronaviruses (CoVs), a genus in the *Coronaviridae* family, order *Nidovirales*, are large, enveloped, RNA viruses that cause highly prevalent diseases in humans and domestic animals. CoVs are spherical enveloped particles about 100–120 nm in diameter with a capped, polyadenylated single-stranded, positive-sense genomic RNA 27.6 to 31 kb in length, the largest known RNA virus genome. The 5′ end of the genome consists of a 65 to 98 nt sequence, termed the *leader* RNA, that is also present at the 5′ end of all subgenomic mRNAs. An untranslated region (UTR) of 200 to 400 nts follows this leader sequence. At the 3′ end of the RNA genome is another UTR of 200 to 500 nts followed by a poly(A) sequence of variable length. Both 5′- and 3′-UTRs are important for RNA replication and transcription. The remaining genomic sequence includes different open reading frames (ORFs) which differ markedly among coronaviruses in number, nt sequence, genes order, and in method of expression. At the 5′ end of each gene, all CoVs have a common intergenic sequence of about 7 bases which is essential for the formation of subgenomic RNAs [1]. 

**Figure 1:**
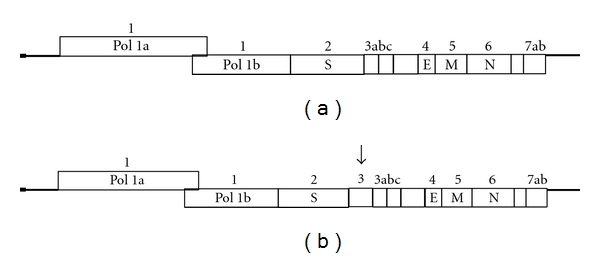
Genetic structure of CCoV type 2 (a) and CCoV type 1 (b). The numbers above the bars indicate ORFs. The names in the bars indicate the protein encoded by the corresponding ORF. The structural proteins are marked by various letters, while the nonstructural proteins are represented by unfilled boxes. The arrow (b) indicates the accessory gene, ORF 3, unique to CCoV type 1.

The first two-thirds of the genome consists of two partially overlapping ORFs, ORF1a and ORF1b. These ORFs are translated into a polyprotein which is the precursor both of the viral RNA-dependent RNA polymerase and of proteases. The one-third in the 3′ end of the genome contains ORFs encoding for the major structural proteins, spike (S), envelope (E), membrane (M), and nucleocapsid (N) proteins. These ORFs are interspersed with several ORFs encoding for different nonstructural proteins, most of which are of unknown function (Figure 1).

The S glycoprotein, which forms the large spikes on the surface, is the major inducer of virus-neutralizing antibodies and plays an important role in the biology and pathogenesis of CCoV infections, inducing both fusion of the viral envelope with host cell membranes and cell-to-cell fusion. In most phylogroup 2 CoVs (*Betacoronavirus*, see below), the 180 kDa S protein is cleaved during or after virus maturation by a cellular protease enhancing the cell fusion activity or viral infectivity. The S protein of phylogroup 1 CoVs (*Alphacoronavirus*, see below) is not cleaved even though some of these viruses can induce cell-to-cell fusion [2]. The small envelope protein E, which is 9–12 kDa, was recently shown to be associated with the viral envelope, and together with the M protein, is required for viral budding [3]. The M membrane protein, a type III glycoprotein, is characteristic in that only a short amino-terminal domain is exposed on the surface of the viral envelope. This domain is followed by a triple-membrane-spanning domain and a large carboxyl-terminal domain inside the envelope [4]. Although the major immunological role has been attributed to the S protein, antibodies to the M protein of MHV can neutralize viral infectivity but only in the presence of complement [5]. The nucleocapsid protein N is a basic phosphoprotein of 50–60 kDa that binds to virion RNA, providing the structural basis for the helical nucleocapsid [6]. N plays a role in viral RNA synthesis and interacts with M protein leading to the formation of virus particles. Additional ORFs encoding nonstructural proteins have been recognized in CoV genomes [7, 8]. The functions of such genes are in most cases unknown and most of them are not essential for virus replication, but may play a part in virulence and host range [9].

Presently, the *Coronaviridae *family is bigeneric and comprises the genera *Coronavirus *and *Torovirus. *In rooted trees, the members of the genus coronavirus consistently form three distinct monophyletic groups, and in pairwise comparisons they form three robust nonoverlapping clusters. This phylogenetic constellation is well recognized, and in publications these three groups are consistently referred to as phylogroups 1, 2, and 3. Phylogroup 1 of the CoVs includes human coronaviruses HCoV-229E and HCoV-NL63, feline coronaviruses (FCoVs) type 1 and type 2, transmissible gastroenteritis virus (TGEV) of swine, porcine respiratory coronavirus (PRCoV), porcine epidemic diarrhea virus (PEDV) and canine coronaviruses (CCoVs). Recently, a ferret coronavirus has been identified as a member of phylogroup 1 [10]. Phylogroup 2 of the mammalian CoVs is split into two subgroups, bovine-like subgroup 2a and SARS-like subgroup 2b. Subgroup 2a includes bovine coronavirus (BCoV), murine hepatitis virus (MHV), sialodacryoadenitis virus (SDAV) found in rats, porcine haemagglutinating encephalomyelitis virus (PHEV), human coronaviruses (HCoV-OC43 and HCoV-HKU1), human enteric coronavirus (HECV-4408), and the newly recognized equine coronavirus (ECoV) [11] and canine respiratory coronavirus (CRCoV) [12]. Soon after its discovery, SARS-CoV was expected to define a new 4th group [13]. However, based on sequence comparisons and the observation that regions of ORF1a of SARS-CoV contain domains that are unique to the group 2 coronaviruses, it has been suggested that it is more directly related to phylogroup 2. Thus, SARS-CoV has been placed within subgroup 2b together with SARS-like CoVs isolated from bats and wild carnivores [14, 15]. A third phylogroup includes the two avian coronaviruses, infectious bronchitis virus (IBV), and turkey coronavirus (TCoV).

**Figure 2:**
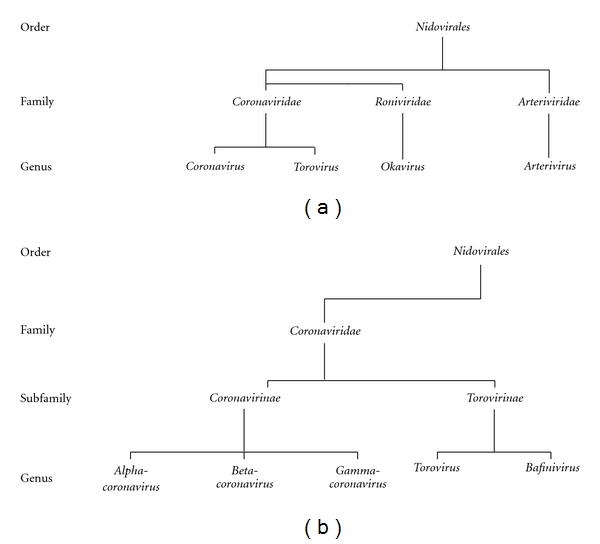
Current taxonomic organization of the *Nidovirales* and of the *Coronaviridae* family (a) and envisaged revision proposed to the ICTV Executive Committee (b).

However, in view of the recent increase in the number of newly discovered coronaviruses and ensuing debates and confusion in the literature concerning coronavirus taxonomy, the members of the coronaviridae study group (CSG) have developed a framework for consistent classification and have established definite genus- and species-demarcation criteria. The *inter*group pairwise amino acid identity scores for the true coronaviruses are comparable to those calculated for structural and nonstructural proteins of different genera in other RNA virus families (e.g., *Potyviridae*, *Picornaviridae*) [16]. The proposed taxonomic revision to the ICTV Executive Committee of the *Coronaviridae *and the organization of the still-to-be-established subfamily *Coronavirinae *are based upon rooted phylogeny and pairwise comparisons using *Coronaviridae*-wide conserved domains in replicase polyprotein (ORF1ab), as well as the structural proteins S, E, M, and N [16–18]. Based on this *de facto *criterion, the CSG has formulated a taxonomic proposal to the ICTV Executive Committee, which would intuitively follow the unofficial (but widely accepted) nomenclature, to convert phylogroups 1 through 3 into genera designated *Alpha*-, *Beta- *and *Gammacoronavirus*, respectively, (Figure 2). The viruses grouped in currently recognized genera form distinct monophylogenetic clusters, but do not share other obvious traits (host tropism, organ tropism, and type of disease), to suggest a common denominator.

## 2. Canine Enteric Coronaviruses: Evolutionary Changes

CCoV was first described during an epizootic in a canine military unit in Germany in 1971 [19]. Starting from this first report, CCoV was isolated repeatedly from affected dogs and today appears to be enzootic worldwide, and dogs of all breeds and ages seem to be susceptible to infection [20–26]. 

Whereas fatal infections are unusual unless mixed with infections by other pathogens or with overcrowding and unsanitary conditions, CCoV alone is an important canine pathogen responsible for epizootics [27]. Nevertheless, the disease caused by CCoV has not been adequately investigated, and the role it plays in canine enteric illness is not established in detail. Since its first isolation in 1971, CCoV has evolved, and in the last two decades researchers have focused on the genomic variability of CCoV strains, identifying new genotypes/types. 

In 2001, by sequence analysis of CCoVs detected in fecal samples collected from diarrheic dogs in the South of Italy, multiple nt substitutions accumulating over a fragment of the M gene were observed [28]. The point mutations affecting the M gene were later observed in the sequences of CCoVs detected in the faeces of two naturally infected pups during the latter stages of long-term viral shedding. These CCoVs are dissimilar from the 1971-like CCoVs, showing an evident genetic drift toward FCoVs type 2, and it was postulated that the two dogs might have been infected by a mixed population of genetically different CCoVs, or that the viruses detected in both pups were the result of mutation/recombination events [29].

These preliminary observations gave a meaningful impulse to studying the genetic evolution of CCoVs. Extensive sequence analysis on multiple regions (ORF1a, ORF1b, and in particular ORF5) of the viral genome from CCoV-positive faecal samples, provided strong evidence for the existence of two separate genetic clusters of CCoV. The first cluster includes CCoVs intermingled with reference CCoV strains, such as Insavc-1 and K378, while the second cluster segregates separately from CCoVs and, presumably, represents a genetic outlier referred to as FCoV-like CCoVs [30]. A possible explanation for this different segregation is that under natural conditions homologous recombination events between highly homologous CoVs, such as CCoV and FCoV, occur frequently. Where the recombination takes place is unknown, but it is known that CCoV is able to use the feline aminopeptidase (fAPN) glycoprotein as a cellular receptor [31] and that, under experimental conditions, cats can be infected with CCoVs [32]. This means that a frequent interspecies circulation either of FCoVs to dogs or of CCoVs to cats may occur, since mixed infections are required to give rise to recombination events. Another supposition is that recombination events have developed in a host other than cat or dog, such as a wild carnivore, or that a wild carnivore might have harbored the immediate ancestor of CCoV. The analysis of CoV RNAs from various wild carnivore isolates could shed light on these hypotheses. According to these speculations, restricted sites of recombination, such as regions of very high nt identity between highly homologous CoVs, may exist in the genome of CoVs. This explanation assumes that CoVs in carnivores possess a sort of “dynamic” genome [30].

**Figure 3:**
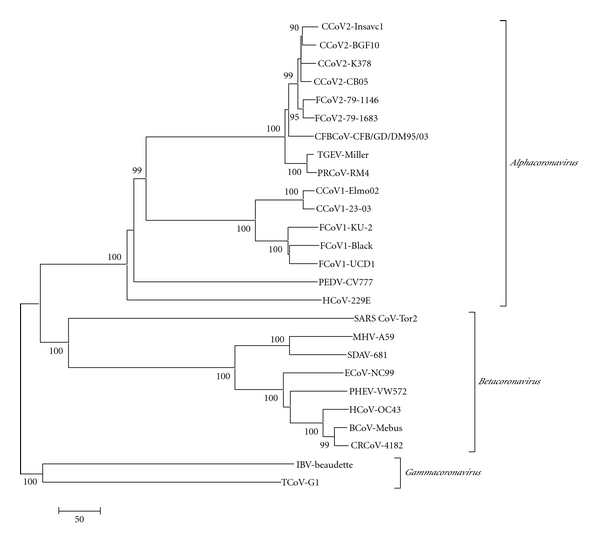
Phylogenetic tree of the spike protein of coronaviruses. CoV strains employed for tree construction are reported (GenBank accession numbers are reported in parentheses). *Alphacoronaviruses*: CCoV type 2 Insavc (D13096), CCoV type 2 K378 (X77047), CCoV type 2 BGF10 (AY342160), CCoV type 2 CB/05 (DQ112226), CCoV type 1 Elmo/02 (AY307020), CCoV type 1 23/03 (AY307021), FCoV type 2 79-1146 (X06170), FCoV type 2 79-1683 (X80799), FCoV type 1 KU-2 (D32044), FCoV type 1 Black (AB088223), FCoV type 1 UCD1 (AB088222), TGEV Miller (S51223), PRCoV RM4 (Z24675), PEDV CV777 (NC_003436), HCoV-229E (NC_002645), CFBCoV CFB/GD/DM95/03 (EF192156). *Betacoronaviruses*: SARS-CoV tor2 (NC_004718), BCoV-Mebus (U00735), CRCoV 4182 (DQ682406), HCoV OC43 (NC_005147), PHEV VW572 (DQ011855), MHV A59 (AY700211), SDAV 681 (AF207551), ECoV NC99 (NC010327). *Gammacoronaviruses*: IBV Beaudette (DQ830981), TCoV G1 (AY342357). Phylogenetic tree was generated by the neighbour-joining method in the Mega3 program [51], and statistical support was provided by bootstrapping >1,000 replicates. Scale bar indicates amino acid substitutions per site. Dog CoVs are grey shaded.

Taking into account the genetic drift toward FCoV observed in the M gene of the FCoV-like CCoVs, the genetic differences between these divergent strains and the typical reference CCoV strains were evaluated in the ORF2 sequence, characterized by a more evident variability. The nt sequence of a region encompassing about 80% of the S gene of one of these FCoV-like CCoVs, strain Elmo/02, clearly indicates that a novel CCoV type, highly divergent from the reference CCoV strains and more closely related to FCoV type 1, circulates among dogs. Comparison of the inferred amino acid sequences revealed about 81% identity to FCoVs type 1 and about 54% identity to both FCoVs type 2 and CCoV reference strains. On the basis of the evident and significant identities between Elmo/02 strain and FCoVs type 1, the new strain Elmo/02 was designated as the prototype of the newly recognised genotype 1 (CCoV type 1), and the reference strains were designated as CCoV type 2 [33]. The high divergence in the amino acid composition and the loss and gain of potential glycosylation sites compared to the most closely related coronaviruses (FCoV type 1, FCoV type 2, and typical CCoV), strongly suggest that the Elmo/02 strain is antigenically poorly correlated to the other coronaviruses of carnivores.  Figure 3 reports the phylogenetic relationship of the S gene from different human and animal CoVs. Phylogenetic analysis clearly demonstrates that CCoV type 1, strain Elmo/02, segregates with FCoVs type 1 rather than reference CCoV type 2 strains and FCoV type 2 strains. Moreover, the presence of the stretch of basic residues RRXRR is indicative of a potential cleavage of the S protein. A similar basic motif is present, approximately in the same position, in all *betacoronaviruses* and *gammacoronaviruses* identified and classified to date. Recently, Lorusso et al. [34] also has described an accessory gene, ORF3, 624 nts in length, unique to CCoV type 1 (Figure 1). The putative encoded protein, with a predicted molecular weight of about 24 kDaa, is 207 amino acids long, and the observation that no transmembrane region has been detected suggests that the protein is secreted from the infected cells. 

The significance of all these data is still unclear, but it raises several questions regarding the biology of CCoVs. The literature on CCoVs-induced disease offers little or no useful information, and although in more recent years additional important data have shed light on the more obscure aspects of the infection, several studies need to be performed to extend knowledge of these two CCoV genotypes. The data acquired on the genome of the enteric CCoVs and on their evolution focus upon important epidemiological outcomes in the field, in terms of both prophylaxis and virus evolution. Hence, the serological correlation between the two viruses requires further study, and several questions regarding the pathobiology of CCoV type 1 and the efficacy of currently available CCoV vaccines that contain only CCoV type 2 remain to be answered.

## 3. Enteric CCoVs: Pathobiology and Epidemiology

The factors regulating the course of the natural diseases caused by enteric CCoVs are not well understood. CCoVs are responsible for enteritis in dogs, and signs of infections may vary from mild to moderate, but they are more severe in young pups or in combination with other pathogens. Common signs include soft faeces or fluid diarrhoea, vomiting, dehydration, loss of appetite, and, occasionally, death. Dual infections by CCoV and canine parvovirus type 2 (CPV2) are especially severe when infections occur simultaneously [35], but CCoVs can also enhance the severity of a sequential CPV2 infection [36].

The natural route of transmission is faecal-oral, and virus in faeces is the major source of infection. In neonatal dogs, the virus appears to replicate primarily in the villus tips of the enterocytes of the small intestine causing a lytic infection followed by desquamation and shortening of the villi and resulting in diarrhoea 18–72 h post infection [37]. Production of local IgAs restricts the spread of the virus within the intestine and arrests the progress of the infection. Therefore, infected dogs may shed virus for as long as 6 months after clinical signs have ceased [29, 38]. 

Recent extensive biomolecular analysis of faecal samples collected from infected dogs in Italy revealed that CCoVs infection is widespread and often characterized by the occurrence of both genotypes simultaneously [39, 40]. CCoVs type 1 and type 2 were found to be common in an Australian animal shelter with CCoV type 1 being prevalent [41]. CCoVs have also been found in Western European dog populations [42]. They have been detected in all European countries examined, and, except for the UK, the prevalence of CCoV type 1 was lower than for CCoV type 2 [25]. Reports of widespread CCoVs have come from Sweden [21] and China [43]. Soma et al. [24] reported that CCoVs are also circulating in Japan, and the detection rate for dogs aged under 1 year was 66.3%, with a simultaneous detection rate of both types up to 40%. 

These data raise several questions, and more indepth investigations into the pathobiology of CCoVs type 1 and type 2 are required. Therefore, failure to isolate CCoV type 1 *in vitro* [40] hinders the acquisition of key information on the pathogenetic role of CCoV type 1 in dogs and prevents an authentic evaluation of the immunological characteristics of this new genotype.

## 4. Divergence Strains and the Emergence of the Pantropic CCoV

CCoVs, like other CoVs, show relatively high mutation frequency, and, in recent decades divergent strains have been described. One of the first observations was from Wesley, [44] who demonstrated that although most CCoV sequences are FCoV-like, the N-terminus of the S gene of CCoV strain UCD-1 is more closely related to TGEV. 

The identification of a novel CCoV, strain UWSMN-1, from a fatal case of gastroenteritis in pups from breeding colonies was reported in Australia in 2001 [23]. Sequence analysis of fragments of the S and polymerase genes confirms that the Australian isolate is divergent from CCoV and FCoV classical strains, and in comparing the 751 nts in the 3′ region of the S gene, it was found that UWSMN-1 had 21 unique sites and that there were 112 sites where the strain was different from at least one of the other strains analyzed. These differences appear to be randomly interspersed, demonstrating that the divergent 5′ region of the S gene in UWSMN-1 is probably not the result of recombination events between FoCVs and CCoVs, as would be indicated if the S gene shared blocks of homology with either FoCV or CCoV S genes. Rather, UWSMN-1 appears to be generally divergent due to a gradual accumulation of mutations throughout its genome, which may be reflective of its isolated evolution in Australia [45].

In 2002, an epizootic outbreak of diarrhea occurred in a Beagle breeding colony in United Kingdom. A new CCoV, strain BGF10, was isolated and characterized. The virus revealed a highly divergent region at the amino-terminal domain of the M protein and a long nonstructural protein 3b of 250 amino acids associated with virulence in other CoVs [46].

In 2005, a fatal disease in puppies was described in Italy, and a pathogenic variant of CCoV, strain CCoV type 2-CB/05, was isolated from all tissues examined except brain [47]. The sequence of the 3′ end of the genome of the pantropic CCoV strain showed a high degree of amino acid identity with CCoV type 2, except for the S protein that displayed the highest identity to FCoV type 2, strain 79-1683 (Figure 3). The highest identity of the E protein was obtained against TGEV strain Purdue in whose cluster the virulent CCoV was found to fall by phylogeny. The N protein too, which was 383 amino acids long, was found to be closely related to TGEV strain Purdue by both sequence analysis and phylogeny. Interestingly, nonstructural protein 3b (22 amino acids) was shorter than expected [48]. In a recent experimental study, Decaro et al. [49] demonstrated that strain CCoV type 2-CB/05 is able to infect dogs seropositive to enteric CCoV and is able to induce clinical signs irrespective of the viral dose administered in the challenged dogs. The infected dogs showed lymphopenia, and viral RNA was detected in thymus, spleen, and lymph nodes of some infected dogs, demonstrating the lymphotropism of the new strain CCoV type 2-CB/05. The study also provides evidence that immunity induced by natural exposure to enteric CCoVs is not fully protective against strain CCoV type 2-CB/05. This important datum highlights the fact that dogs vaccinated with enteric CCoV may acquire subclinical infections with CCoV type 2-CB/05-like virus, resulting in lymphopenia and predisposing for opportunistic pathogens and/or for a more severe disease induced by canine parvovirus.

Recently, CCoVs with a potential double recombinant origin through partial S-gene exchange with TGEV were identified in the gastrointestinal tract and internal organs of pups which had died of acute gastroenteritis [50]. A TGEV-like CCoV, strain UCD1, has been previously described, but only partial S-gene sequences were determined, thus preventing a complete genomic characterization [44]. The new CCoV strains were strictly related to TGEV in the N-terminal domain of the spike protein, whereas the rest of the genome revealed a higher genetic relatedness to CCoV type 2 isolates. The relevant antigenic differences observed between reference CCoV type 2 and recombinant TGEV-like CCoVs could have implications for prophylaxis programs, as dogs administered classical CCoV vaccines may be susceptible to infection caused by the recombinant virus.

## 5. Respiratory Canine Coronavirus

Another example of the evident evolution of dog coronaviruses, as a consequence of the accumulation of point mutations, small insertions, and deletions in coding and non-coding regions of the genome, is the recent identification of a novel coronavirus, canine respiratory coronavirus CRCoV, in tissue samples collected from the respiratory tract of diseased dogs. During a survey to establish the causes of canine infectious respiratory disease in a large rehoming kennel in the United Kingdom, a CRCoV was isolated in tracheal and lung samples in dogs with mild clinical symptoms. The virus showed a close relationship to the *betacoronavirus* in the polymerase and S genes, with the highest amino acid identity with the corresponding BCoV proteins and proved to be only distantly related to enteric CCoVs. By sequence comparison of cDNA polymerase in the analyzed 251 bp sequence, the identity was 98.8% for BCoV and 98.4% for HCoV polymerase gene, whereas it was only 68.5% for CCoV, strain 1–71. When comparing the amino acid sequence obtained by translation of the cDNA sequence from CRCoV to the amino acid sequence of BCoV, HCoV-OC43, and enteric CCoV spike proteins in an overlap of 1093 amino acids, the identities were 96%, 95.2%, and 21.2%, respectively (Figure 3). Moreover, the presence of the HE gene, which is a characteristic protein gene of the members of the *betacoronaviruses*, has been demonstrated in the CRCoV genome [12, 52]. Since its detection in 2003, CRCoV has been found to be present in dogs in other European countries, as well as in Canada and in Japan [53–58]. The evolution of the *betacoronaviruses* is closely linked, and it has been suggested that these viruses share a recent common ancestor. CRCoV may also share this ancestor or may have originated from a transfer of BCoV to dogs. In order to conclusively answer the question of whether CRCoV has only recently emerged, a greater number of archived materials need to be tested. In addition, more sequence information from CRCoV strains will be required to perform phylogenetic analyses that may shed more light on their origins.

## 6. New Advances in Diagnosis

The clinical signs most frequently associated with enteric CCoVs are not easily differentiated from those associated with other enteric pathogens such as CPV2 or canine rotavirus and canine adenovirus. Consequently, CCoV diagnosis requires laboratory confirmation. The diagnostic techniques employed for the detection of CCoVs in fecal samples include electron microscopy (EM), virus isolation (VI) in cell cultures, and biomolecular analysis. EM examination of negatively stained fecal suspensions and immune electron microscopy are rapid procedures for detecting coronavirus and appear to be valuable diagnostic tools [59]. However, *coronavirus-like* particles in intestinal contents often resemble coronaviruses [60], and EM examination required specialized laboratories and technicians. VI is the most commonly used technique for diagnosis of CCoVs infection [45], but is more complex, more time-consuming and less sensitive than other methods. CCoV type 2 grows on several cell lines of canine and feline origin, and the identification of an isolate requires neutralization of the cytopathic effects and/or immunofluorescence test with a reference serum or monoclonal antibodies. Failure to isolate CCoV type 1 in cell cultures [40] reduces the changes of identifying many forms of enteritis caused by this virus, so the frequency of CCoV type 1 disease is probably underestimated. Such difficulties and limitations prevent an authentic evaluation of the immunological characteristics of this new genotype and hinder the acquisition of key information on its pathogenetic role in dogs. 

In the past decade, several PCR-based methods have been developed for detecting CCoV RNA in the feces of dogs, allowing the detection limits of virus isolation to be overcome. PCR has been identified as the gold standard because of the improvement in both sensitivity and specificity when compared to conventional methods [20, 23, 61, 62]. Therefore, none of the developed PCRs were designed to be quantitative. Moreover, conventional PCR contains a certain risk of carryover contamination due to post-PCR manipulations and to a second amplification step in nested PCR systems, especially when a high sample throughput is required. Conversely, real-time TaqMan RT-PCR enables a sensitive and specific quantitation of viral RNA [63–66]. Decaro et al. [67] developed a real-time fluorogenic RT-PCR, a simple, rapid, and reproducible method for the detection and quantitation of CCoV RNA in the feces of infected dogs, based on the TaqMan technology. In comparison to conventional RT-PCR, the fluorogenic assay is a closed system in which the tube is never opened postamplification, ruling out the possibility of cross-contamination. The main advantage of the fluorogenic dye system consists of quantifying CCoV RNA amounts in fecal samples with a high degree of reproducibility and precision compared to quantitative gel-based PCR assays [68, 69]. 

Recently, two genotype-specific fluorogenic RT-PCR assays were developed for the detection, discrimination, and quantitation of CCoV type 1 and CCoV type 2 RNAs in the feces of dogs with diarrhea. The assay showed high specificity, sensitivity, and reproducibility, allowing a precise quantitation of CCoVs RNA over a linear range of about eight orders of magnitude, from 10^1^ to 10^8^ copies of standard RNA. The genotype-specific fluorogenic assay can be useful to detect and measure viral loads in fecal samples collected from dogs naturally or experimentally infected with type 1, type 2, or both genotypes [39].

## 7. Conclusion

One of RNA's most intriguing features is its ability to carry genetic information despite its labile nature. CoVs are unique among RNA viruses in many aspects of their biology. They are characterized by a extremely large genome, by a nested set of subgenomic mRNAs, by a discontinuous transcription mechanism, and by a high frequency of RNA recombination events because of the high error frequencies of RNA polymerase [70]. Genetic recombination is an important mechanism for generating novel genomes that may have selective advantages over parental genomes. In the evolution of RNA viruses, RNA recombination is a widespread phenomenon that has shaped viruses by rearranging viral genomes or disseminating functional modules among different viruses [71]. Although nonsegmented genomes of RNA viruses generally exhibit very low or undetectable recombination frequencies, the recombinations for the entire CoV genome have been calculated to be as high as 25% [72]. The high frequency of RNA recombination in CoVs is probably the result of the unique mechanism of coronavirus synthesis, which involves discontinuous transcription and polymerase jumping. It is possible that the viral polymerase associated with the incomplete nascent RNAs, dissociates from its template at a random point, and switches to a homologous site on a different RNA template to complete RNA synthesis by a copy-choice mechanism [73]. Depending on the precision of the repair mechanism, the repaired genome may be similar to the parental genome, or it may contain further mutations. This illustrates that sequence diversity in RNA sequences generated by genetic recombination can involve both gross changes and minor mutations. Genetic divergence within the *alphacoronavirus* is accounted for by linear evolution as well as by sudden dramatic shifts generated by RNA deletions or recombination [74].

RNA recombination has been developed into a potent genetic tool to introduce desired RNA sequences into CoV genomes, revealing itself as an important mechanism in the natural evolution of CoVs. For example, new strains of IBV in poultry flocks are the results of natural recombination between different field strains. FCoVs type 2 have arisen by recombination events between FCoV type 1, wholly feline, and CCoV, resulting in a FCoV genome consisting of the spike gene and part of the adjacent gene from CCoV [75]. Recombination may also play a role in the evolution of different coronavirus species and may also explain the acquisition of the HE gene from an mRNA of influenza C virus by a progenitor of the *betacoronaviruses* [1]. The S protein of PEDV occupies an intermediate position between HCoV 229E and TGEV [76], while the S protein of PRCoV is highly related to TGEV but has a large deletion in the N-terminus (more than 200 amino acids) that may explain the change in the pathobiology of the virus [77]. SARS emerged as a human disease associated with pneumonia in Guangdong Province, China, in November 2002. The etiological agent of SARS appears to be an animal virus that crossed to humans. The existence of a 29-nt deletion sequence in the genome of a wild mammal CoV confirms the adaptation of this new virus to humans [78]. 

As a consequence of the relatively high mutation frequency, the RNA viruses have the potential to rapidly adjust to certain negative pressures, such as those presented by the immune system. Recombination events affecting CCoVs could clarify the evolutionary processes leading to the proliferation of new virus strains, serotypes, and subtype, as happened for SARS-CoV and for strain CCoV type 2-CB/05. Notwithstanding the several studies carried out on CCoVs, there are a lot of aspects yet to be clarified: the meaning of simultaneous infection by CCoV type 1 and CCoV type 2, the real pathogenetic role of these two viruses, the immune response against CCoV type 1 and CCoV type 2, and the assessment of CCoV type 2 CB/05-like virus distribution among dog populations.
